# Quality of life and its contributors among patients with homozygous familial hypercholesterolemia in China

**DOI:** 10.3389/fpubh.2025.1642895

**Published:** 2025-08-27

**Authors:** Mi Tang, Ziping Ye, Xueyan Zhang, Jiangjiang He, Qi Kang, Zhenqing Tang, Luya Wang, Ya Yang, Yan Yang, Shanlian Hu

**Affiliations:** ^1^Shanghai Health Development Research Center (Shanghai Medical Information Center), Shanghai, China; ^2^School of Public Administration, Hainan University, Hainan, Haikou, China; ^3^Department of Health Policy, School of Medicine, Stanford University, Stanford, CA, United States; ^4^Shanghai Municipal Center for Health Promotion, Shanghai, China; ^5^Beijing Anzhen Hospital, Beijing Institute of Heart, Lung and Blood Vessel Diseases, Capital Medical University, Beijing, China; ^6^School of Public Health, Fudan University, Shanghai, China

**Keywords:** China, epidemiological survey, homozygous familial hypercholesterolemia, quality of life, rare disease

## Abstract

**Background:**

China is one of the countries with the largest number of patients with homozygous familial hypercholesterolemia (HoFH) in the world. Improving the quality of life and health outcomes for HoFH patients in China is of great importance. Therefore, the aim of this study is to assess the quality of life of HoFH patients in China and to investigate the factors that influence quality of life.

**Methods:**

Data were obtained from a national epidemiological survey of HoFH patients conducted by Beijing Anzhen Hospital, affiliated with Capital Medical University, during 2017–2019. The questionnaire included patient demographic information, disease information, family economic status and health-related quality of life. Quality of life was assessed using the EuroQol five-dimension three-level (EQ-5D-3L) questionnaire. Data processing and statistic tests are performed using Python libraries.

**Results:**

This investigation incorporated a sample size of 53 patients diagnosed with HoFH, with an average age of 27.92 years. It was observed that 45.28% of these patients' families were subjected to catastrophic health expenditure. The mean and median health utility scores were calculated to be 0.849 and 0.875, respectively, a figure that is significantly lower when compared to the scores of the general population. Furthermore, it was noted that 52.83% of the patients exhibited some level of difficulty or extreme difficulty in at least one dimension of the EQ-5D-3L. Significant Factors negatively affecting the quality of life of HoFH patients include the presence of atherosclerotic cardiovascular disease (ASCVD), hospitalisation in the past year, taking multiple medications, higher than average healthcare expenditure per capita, catastrophic healthcare expenditure and debt due to HoFH. After application of Benjamini-Hochberg method to minimize type 1 error, the occurrence of ASCVD and debt due to HoFH are likely to exert the most significant influence. Substantial relevance of the two factors are also evidenced by large effect sizes and adequate statistical power.

**Conclusions:**

Chinese patients with HoFH showed lower quality of life than the general population. Policy makers should consider the policy that improves early diagnosis, employment prospects, and the availability of HoFH-related interventions and provision of financial assistance for patients suffering from HoFH.

## 1 Introduction

Homozygous familial hypercholesterolemia (HoFH) is a rare, inherited disease caused by homozygous or compound heterozygous mutations in key genes that play a critical role in the metabolism of low-density lipoprotein cholesterol (LDL-C) (1). The genetic mutations in HoFH patients result in a decrease or loss of plasma low-density lipoprotein clearance ability, leading to a significant elevation of LDL-C levels that are 6–8 times higher than normal individuals from birth (2). Sustained exposure to elevated levels of LDL-C can potentially result in cholesterol accumulation within the arterial walls. This accumulation can subsequently lead to the development of atherosclerotic plaques, thereby considerably escalating the risk of atherosclerotic cardiovascular diseases (ASCVD). These conditions encompass acute myocardial infarction and sudden death, which predominantly manifest during adolescence. Without any intervention, patients usually succumb to coronary heart disease before reaching the age of 30 (3, 4).

Recent molecular genetic studies have indicated that the global prevalence of HoFH ranges from 1/300,000 to 1/170,000 (5–7). However, the prevalence rates of this disease vary across different regions and ethnic groups. For instance, the Hokuriku region of Japan has a prevalence rate of 1/1,450,000 (8), while the rate is 1/860,000 in Germans (9), 1/275,000 in Quebec French Canadians (10), 1/450,000 in Spaniards (11), and 1/300,000 in the Dutch (12). In certain ethnic groups within specific regions, such as Afrikaners in South Africa, the prevalence rate is higher, at 1/30,000 (13). The “Chinese Expert Consensus on Screening and Diagnosis of Familial Hypercholesterolemia” indicates the prevalence rate of HoFH in China to be between 1/1,000,000 and 3/1,000,000, with an estimated 1,408–4,224 HoFH patients in mainland China by the end of 2024 (14). In Taiwan, the “Health Promotion Administration, Ministry of Health and Welfare” reported 67 identified cases of HoFH patients since the implementation of the Rare Diseases Prevention and Treatment Act between February 2000 and April 30, 2025 (15). The primary treatment methods for HoFH involve lifestyle modifications (e.g., dietary adjustments, weight loss), medications, plasma exchange, liver transplantation, and other surgical interventions (16, 17). Among these, medication remains the primary method of reducing LDL-C levels, including statins, ezetimibe, proprotein convertase subtilisin/kexin type 9 (PCSK9) inhibitors, evinacumab, lomitapide, and mipomersen for more severe cases (18). Notwithstanding the progress made in comprehending the intricacies of HoFH disease, individuals afflicted with this condition persistently grapple with substantial challenges arising from deferred diagnosis and insufficient treatment. These burdens incorporate not only the physical distress induced by the disease, but also the unpredictability concerning prognosis, social, psychological, and economic stressors. These factors culminate in suboptimal health outcomes and a decreased quality of life for patients (19). Given the scarcity of data, research on the quality of life of HoFH patients remains constrained.

In May 2018, HoFH was included in the first group of rare diseases catalog in China (20), which means policymakers have noticed the insufficient support of patients with HoFH. However, liver transplantation and lipoprotein apheresis (LA) are not widely accessible, and medication and lifestyle modifications remain the primary interventions for patients with HoFH. Nonetheless, conventional statin medications often fail to meet treatment objectives, while PCSK9 inhibitors, which can further decrease LDL-C levels, are costly. China is one of the countries with the largest number of HoFH patients globally. Improving the quality of life and health outcomes for HoFH patients in China is therefore of importance, and it is crucial to understand the factors that determine their quality of life. Thus, the main goals of this research are 2-fold: to assess the quality of life of HoFH patients in China, and to investigate the factors that influence the quality of life of HoFH patients.

## 2 Materials and methods

### 2.1 Sampling methods

Since the epidemiological information regarding HoFH patients is still unclear in China and there is no complete sample framework available. This study relies on the Chinese familial hypercholesterolemia clinic screening project conducted by Beijing Anzhen Hospital, affiliated with Capital Medical University (21). The study used a convenience sampling method to select research subjects based on medical records and voluntary clinic activities at Beijing Anzhen Hospital.

### 2.2 Procedure

To conduct this survey, researchers interviewed patients in person. Doctors at Beijing Anzhen Hospital used the Dutch Lipid Clinic Network (DLCN) diagnostic criteria (22), to identify FH patients, and diagnosed HoFH patients using diagnostic criteria from the European Atherosclerosis Society (1). The study focused on HoFH patients who had been diagnosed between September 2017 and May 2019. Patients with heterozygous familial hypercholesterolemia (HeFH), sitosterolemia, or who had not yet been diagnosed with HoFH were excluded. The study protocol was approved by the Beijing Anzhen Hospital Ethics Committee (Approval No. 2017035).

Before starting the official survey, investigators conducted a preliminary survey on-site to refine the questionnaire. Patients were fully informed about the survey and signed an informed consent form before participating in face-to-face interviews. Patients who had multiple visits or participated in the voluntary clinic were not surveyed more than once. The survey data were entered by two people and cross-checked for discrepancies. Any logical errors or omissions were resolved by contacting patients by phone for further clarification.

### 2.3 Questionnaires

To conduct the survey, a standardized questionnaire called “Quality of Life in Homozygous Familial Hypercholesterolemia Patients” was used. This questionnaire collected patients' demographic information (such as age and gender), disease-related details (such as when the disease was diagnosed and how long it has lasted), family economic status (including income and medical expenses), and health-related quality of life.

Patient health-related quality of life was evaluated using the EuroQol five-dimension three-level (EQ-5D-3L) questionnaire, which has two parts: the EuroQol five-dimension (EQ-5D) descriptive system and the EQ visual analogue scale (EQ-VAS). The EQ-5D-3L descriptive system has five dimensions: mobility, self-care, usual activities, pain or discomfort, and anxiety or depression, with three levels each: no problems, some problems, and extreme problems. EQ-VAS is a vertical scale where patients rate their health status from 0 to 100, with 100 representing the best possible health and 0 representing the worst (23).

The Chinese EQ-5D-3L algorithm (24) was used to calculate patients' health utility value based on their responses to the questionnaire's dimensions and levels. Nonetheless, due to the absence of an EQ-5D Youth version (EQ-5D-Y) value set for China at the time of the questionnaire's development, the study was restricted to participants aged 12 and above.

### 2.4 Data analysis

The process of data cleaning and statistical analysis was implemented through Python 3.10.8. The first step is Shapiro-Wilk normality test for each combination of independent variable and dependent variable. The following step is assigning appropriate correlation statistical test for each combination based on result of previous step and minimum sample size of levels. Since no combination passes normality test, in addition, all minimum sample sizes are < 30 for all the combinations, non-parametric tests like Mann-Whitney U test and Kruskal–Wallis test are applied for correlation analysis for 2 and 3 levels cases, respectively (Supplementary Tables S1, S2). To minimize type I errors risk due to multiple tests, Benjamini-Hochberg Procedure is applied at dual thresholds—maintaining stringent control (α = 0.05) for primary findings while permitting exploratory analysis (α = 0.10) for hypothesis generation, which is the expected proportion of false positives among the rejected hypotheses.

For all tested associations, effect sizes, statistic power, a priori sample size estimation are further quantified (Supplementary Tables S4–S5). Rank-biserial correlation rrb and tie-corrected epsilon-squared ε^2^ are employed as effect sizes for binary, multiclass features respectively. *Post-hoc* statistic power is calculated based on observed cohort. The minimum sample size that ensures statistic power ≥ 80% is computed for each feature by *t*-test/ANOVA approximation under equal group sizes assumption.

## 3 Results

### 3.1 Descriptive statistics

**Figure 1:**
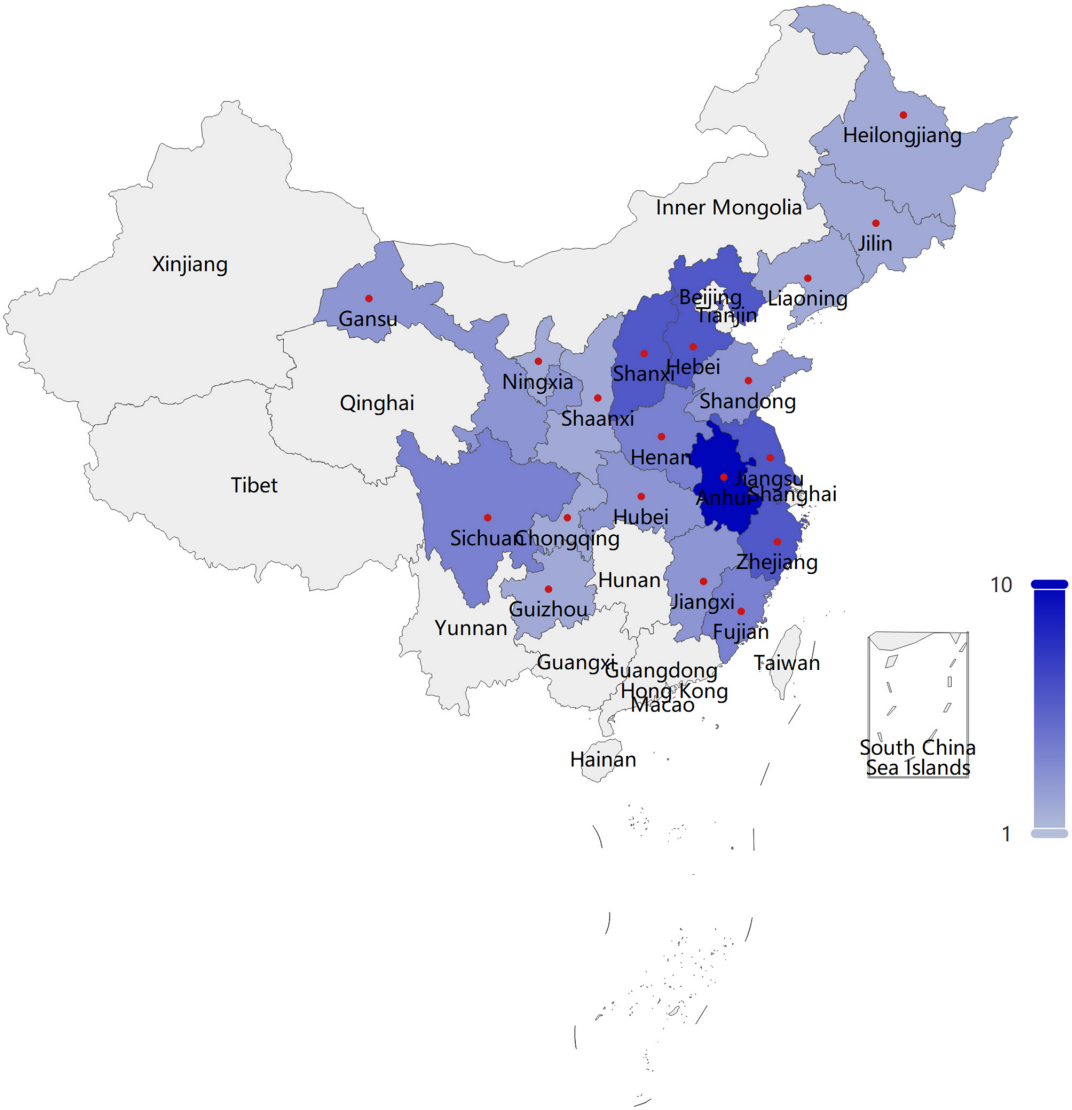
Regional distribution of 53 HoFH patients.

This research incorporated a sample of 53 patients diagnosed with HoFH, originating from 19 provinces or regions (Supplementary Table S5), inclusive of Anhui, Hebei, Jiangsu, Shanxi, Zhejiang, etc., as depicted in Figure 1 (The boundaries of this map are derived from PyECharts and align with the official standards of China). The mean age of the patients was calculated to be 27.92 years, with the age distribution spanning from 12 to 54 years. Over fifty percent of the patients have been diagnosed with HoFH for a duration exceeding 5 years, with approximately 30.19% of these individuals having been diagnosed for a minimum of 10 years. A significant proportion of the patients, specifically 69.81%, have been living with this disease for a decade or longer. Within the sample of 53 patients, 30 individuals had a history of ASCVD, and an equal number exhibited LDL-C values exceeding 10 mmol/L. The severity of HoFH was classified using the “Severe Familial Hypercholesterolemia” criteria as set forth by the International Atherosclerosis Society (IAS) (25). It was determined that a minimum of 40 patients, representing 75.47% of the total sample, were categorized as having severe HoFH.

A comparative analysis was conducted between the economic condition of the families of the 53 patients and the income and expenditure of urban and rural inhabitants in 2018, utilizing data from the National Bureau of Statistics of China. The disposable income per capita of urban and rural residents was reported to be 39,251 and 14,617 Chinese yuan (CNY), respectively. Moreover, the per capita expenditure on healthcare and medical services for urban and rural residents was recorded as 2,046 yuan and 1,240 yuan, respectively (26). The findings indicated that the disposable income per capita of 18 families of patients, constituting 33.96%, surpassed the national average. Furthermore, the healthcare expenditure of 42 families of patients, representing 79.25%, was observed to exceed the national average. Additionally, a significant 45.28% of the patient's families underwent catastrophic health expenditure, while 49.06% accumulated debt as a result of HoFH. This signifies a substantial economic strain imposed by the disease on both patients and their family members. The fundamental details pertaining to the sample are illustrated in Table 1.

**Table 1:** Descriptive information of 53 HoFH patients.

**Items**			**Numbers**	**Percentage (%)**
Demographic characteristics	Gender	Male	26	49.06
		Female	27	50.94
	Age	12–19	12	22.64
		20–29	19	35.85
		≥30	22	41.51
	Residence type	Rural	30	56.60
		Urban	23	43.40
	Marital status	Unmarried	32	60.38
		Married	21	39.62
	Employment status	Employed^a^	23	43.40
		Unemployed	12	22.64
		Student	18	33.96
Disease-related characteristics	Diagnosis time	0–4 years	24	45.28
		5–9 years	13	24.53
		≥10 years	16	30.19
	Disease duration	0–9 years	16	30.19
		10–19 years	21	39.62
		≥20 years	16	30.19
	ASCVD occurrence	No	23	43.40
		Yes	30	56.60
	LDL-C	≤ 10 mmol/L	23	43.40
		>10 mmol/L	30	56.60
	Hospitalization in the past year	No	27	50.94
		Yes	26	49.06
	Number of medications	< 5	33	62.26
		≥5	20	37.74
Family economic characteristics	Does the disposable income per capita of the family exceed the average value?	No	35	66.04
		Yes	18	33.96
	Does the healthcare expenditure per capita of the family exceed the average value?	No	11	20.75
		Yes	42	79.25
	Has catastrophic health expenditure occurred in the family?^b^	No	29	54.72
		Yes	24	45.28
	Is there debt due to HoFH in the family?	No	27	50.94
		Yes	26	49.06

ASCVD, atherosclerotic cardiovascular diseases; LDL-C, low-density lipoprotein cholesterol; HoFH, homozygous familial hypercholesterolemia.

^a^Employment status includes flexible employment.

^b^Catastrophic heath expenditure occurs when a household's total out-of-pocket health payments equal or exceed 40% of household's capacity to pay or non-subsistence spending, household's capacity to pay is calculated by subtracting household food expenditure from household consumption expenditure when food expenditure is lower than subsistence spending (27).

### 3.2 Distribution of EQ-5D dimensions

In terms of the distribution of EQ-5D dimensions, it was found that 25 individuals with HoFH did not report any difficulties across all five dimensions of the EQ-5D questionnaire. A particularly high number of patients (88.68%) reporting no difficulties in the self-care dimension. However, 52.83% of patients reported experiencing some degree of difficulty or extreme difficulty in at least one dimension. The dimensions of pain/discomfort, anxiety/depression, and usual activities were identified as the most problematic, with 39.62%, 32.08%, and 30.19% of patients experiencing difficulties in each dimension, respectively. Further details can be found in Table 2.

**Table 2:** Distribution of EQ-5D dimensions in 53 HoFH patients.

**Dimensions**	**No problems**		**Some problems**		**Extreme problems**	
	**Numbers**	**Percentage (%)**	**Numbers**	**Percentage (%)**	**Numbers**	**Percentage (%)**
Mobility	40	75.47	13	24.53	0	0.00
Self-care	47	88.68	6	11.32	0	0.00
Usual activities	37	69.81	15	28.30	1	1.89
Pain or discomfort	32	60.38	21	39.62	0	0.00
Anxiety or depression	36	67.92	15	28.30	2	3.78

### 3.3 Analysis of health utility value and VAS score

Table 3 displays that the average health utility of 53 HoFH patients was 0.849, and the mean VAS score was 71.98. There were no statistically significant differences in health utility values and VAS scores of HoFH patients in terms of gender, age, residence type, marital status, employment status, disease diagnosis time, disease duration, LDL-C value, and whether the disposable income per capita in the household exceeded the average level. Statistically significant differences in patients' health utility values were found in relation to whether they had experienced ASCVD, been hospitalized in the past year, number of medications, healthcare expenditure per capita exceeding the average, catastrophic health expenditure, and debt due to HoFH (*p* < 0.05). Factors that reveal statistically significant differences (*p* < 0.05) in VAS scores distribution among levels include whether they had experienced ASCVD and debt due to HoFH. Patients with HoFH who had experienced ASCVD had a lower average health utility value of 0.779 and VAS score of 66.00 compared to those who had not experienced ASCVD. Patients who had been hospitalized in the past year also had lower mean health utility values (0.788) and VAS scores (67.50) than those who had not been hospitalized. Patients who were taking five or more types of medication had lower mean health utility values and VAS scores. Patients with higher family healthcare expenditure, catastrophic health expenditure, or debt due to HoFH all had lower mean health utility values and VAS scores.

**Table 3:** Health utility values and VAS scores of patients with HoFH.

**Features and *p* values**	**Groups**	**Health utilities**				**VAS**			
		**Mean**	**SD**	**Median**	**Quartile**	**Mean**	**SD**	**Median**	**Quartile**
Whole sample		0.849	0.178	0.875	0.770	71.98	17.88	75.00	60.00
Gender	Male	0.881	0.154	1.000	0.790	73.65	20.62	77.50	61.25
*P* (raw and BH-adjusted)	Female	0.819	0.197	0.862	0.697	70.37	15.00	70.00	60.00
		0.228	0.490			0.358	0.506		
Age	12–19	0.901	0.135	1.000	0.783	75.83	16.63	80.00	60.00
	20–29	0.865	0.169	0.875	0.795	72.11	15.93	70.00	60.00
	≥30	0.808	0.203	0.795	0.690	69.77	20.38	72.50	52.50
*P* (raw and BH-adjusted)		0.412	0.537			0.652	0.758		
Residence type	Rural	0.854	0.167	0.938	0.709	73.17	17.14	75.00	60.00
	Urban	0.844	0.195	0.875	0.792	70.43	19.06	75.00	60.00
*P* (raw and BH-adjusted)		1.000	1.000			0.737	0.789		
Marital status	Unmarried	0.873	0.154	0.938	0.783	73.75	16.61	75.00	60.00
	Married	0.813	0.209	0.869	0.684	19.77	69.29	75.00	50.00
*P* (raw and BH-adjusted)		0.336	0.506			0.478	0.598		
Employment status	Employed	0.870	0.189	1.000	0.795	72.61	20.22	75.00	55.00
	Unemployed	0.799	0.170	0.789	0.706	68.75	13.84	75.00	60.00
	Student	0.857	0.172	0.938	0.773	73.33	17.74	72.50	60.00
*P* (raw and BH-adjusted)		0.351	0.506			0.677	0.758		
Diagnosis time	0–4 years	0.864	0.171	1.000	0.755	73.54	20.98	80.00	60.00
	5–9 years	0.879	0.209	1.000	0.862	75.00	14.29	80.00	70.00
	≥10 years	0.803	0.164	0.795	0.693	67.19	15.27	67.50	50.00
*P* (raw and BH-adjusted)		0.290	0.506			0.310	0.506		
Disease duration	0–9 years	0.914	0.143	1.000	0.852	79.38	16.52	85.00	67.50
	10–19 years	0.826	0.199	0.869	0.770	69.05	18.95	70.00	60.00
	≥20 years	0.816	0.175	0.795	0.684	68.44	16.50	70.00	50.00
*P* (raw and BH-adjusted)		0.175	0.403			0.116	0.289		
ASCVD occurrence	No	0.942	0.094	1.000	0.875	79.78	14.73	80.00	70.00
	Yes	0.779	0.196	0.786	0.629	66.00	17.98	67.50	50.00
P (raw and BH-adjusted)		0.001	0.018			0.006	0.056		
LDL-C	≤ 10 mmol/L	0.869	0.180	1.000	0.752	72.83	16.57	70.00	60.00
	>10 mmol/L	0.835	0.178	0.829	0.773	71.33	19.07	77.50	60.00
*P* (raw and BH-adjusted)		0.367	0.506			0.906	0.937		
Hospitalization in the past year	No	0.909	0.116	1.000	0.829	76.30	15.97	80.00	62.50
	Yes	0.788	0.210	0.795	0.610	67.50	18.93	70.00	50.00
*P* (raw and BH-adjusted)		0.037	0.157			0.089	0.242		
Number of medications	< 5	0.897	0.132	1.000	0.795	76.06	15.90	80.00	60.00
	≥5	0.770	0.217	0.792	0.596	65.25	19.30	67.50	50.00
*P* (raw and BH-adjusted)		0.029	0.145			0.051	0.170		
Does the disposable income per capita of the family exceed the average value?	No	0.829	0.178	0.875	0.709	70.86	17.88	75.00	55.00
	Yes	0.888	0.132	0.938	0.795	74.17	16.20	75.00	61.25
*P* (raw and BH-adjusted)		0.371	0.506			0.683	0.758		
Does the healthcare expenditure per capita of the family exceed the average value?	No	0.968	0.073	1.000	1.000	77.27	17.23	80.00	67.50
	Yes	0.818	0.185	0.829	0.699	70.60	17.98	70.00	60.00
*P* (raw and BH-adjusted)		0.010	0.073			0.281	0.506		
Has catastrophic health expenditure occurred in the family?	No	0.908	0.130	1.000	0.862	76.55	15.53	80.00	70.00
	Yes	0.778	0.204	0.792	0.666	66.46	19.25	67.50	50.00
*P* (raw and BH-adjusted)		0.016	0.096			0.060	0.179		
Is there debt due to HoFH in the family?	No	0.926	0.119	1.000	0.872	77.04	15.08	80.00	70.00
	Yes	0.770	0.196	0.786	0.618	66.73	19.28	67.50	50.00
*P* (raw and BH-adjusted)		0.001	0.018			0.046	0.170		

ASCVD, atherosclerotic cardiovascular diseases; LDL-C, low-density lipoprotein cholesterol; HoFH, homozygous familial hypercholesterolemia.

Statistically significant features are identified by comparing ascending raw *p*-values against their Benjamini-Hochberg critical thresholds, setting the largest raw *p*-value (*p* = 0.0012 in this cohort) that is less than its threshold as baseline, rejecting all null hypotheses of equal or smaller raw *p*-values. The critical threshold is α × i/m, where α is the control level of false discovery rate (FDR), i is the rank and m is the total number of tests, i.e., 30. Significance can also be identified by another mathematically equivalent method, namely rejecting all null hypotheses with Benjamini-Hochberg adjusted *p*-values below α, both method yields same results. For transparency, both raw (left) and adjusted *p*-values are reported in the analysis and listed in Table 3.

At the conventional false discovery rate threshold (α = 0.05), both ASCVD status and debt related to HoFH showed statistically significant associations with health utility scores (all adjusted *p*-values < 0.05). In contrast, no significant correlates were identified for VAS scores at this stringent threshold. When employing a more lenient exploratory threshold (α = 0.10), additional associations emerged: health utility scores were nominally associated with both healthcare expenditure per capita exceeding the average and catastrophic health expenditure, while ASCVD additionally demonstrated marginal significance with VAS scores. These secondary findings should be interpreted with caution as hypothesis-generating observations that require replication in independent cohorts.

### 3.4 Analysis of *p*-values, effect size, statistic power

Although statistical significance (*p* < 0.05) was observed, interpreting results based solely on *p*-values may be insufficient, as *p*-values do not reflect effect sizes or the robustness of findings. Therefore, effect sizes and statistical power analyses are further integrated to provide a more comprehensive evaluation of the results.

**Figure 2:**
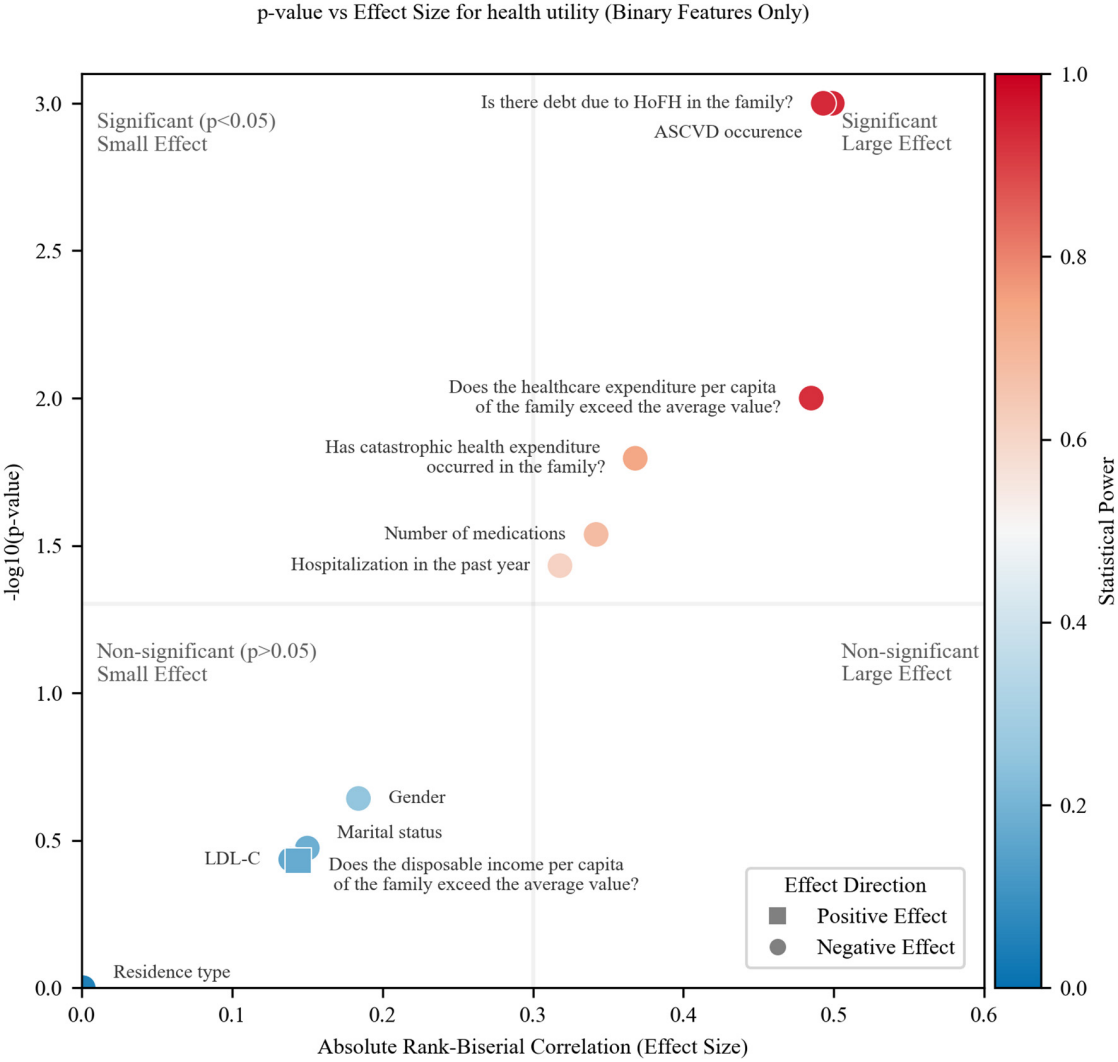
Effect size, significance and statistical power for binary features and health utility.

Figure 2 presents a comprehensive visualization of binary feature associations (health utility), combining effect magnitude (absolute rank-biserial correlation, |r_rb_|), statistical significance (–log_10_*p*), and statistical power (color gradient). The plot is partitioned by threshold lines at *p* = 0.05 (horizontal) and |r_rb_| = 0.3 (vertical), defining four regions: significant (*p* < 0.05) and large effect (|r_rb_| > 0.3) (top-right), significant but small effect (top-left), non-significant but large effect (bottom-right), non-significant and small effect (bottom-left).

All features clustered exclusively in either significant/large-effect quadrant (top-right) or non-significant/small-effect quadrant (bottom-left). No features showed contradictory patterns, such as statistical significance with negligible effect sizes, indicating a clear separation in the associations. ASCVD, debt due to HoFH, and above-average healthcare expenditure demonstrated large effects, small *p*-values, robust power (>0.8, red hues).

The absence of features in the bottom-right quadrant (large but non-significant effects) implies that: for the observed effect sizes, the sample size was sufficient to detect statistically significant associations when they reached the ‘large effect' threshold. However, this does not guarantee adequate power for smaller but still meaningful effects. Power gradients (red to blue) highlight features requiring larger sample sizes for definitive analysis (e.g., low-power points in the bottom-left region) by blue color.

**Figure 3:**
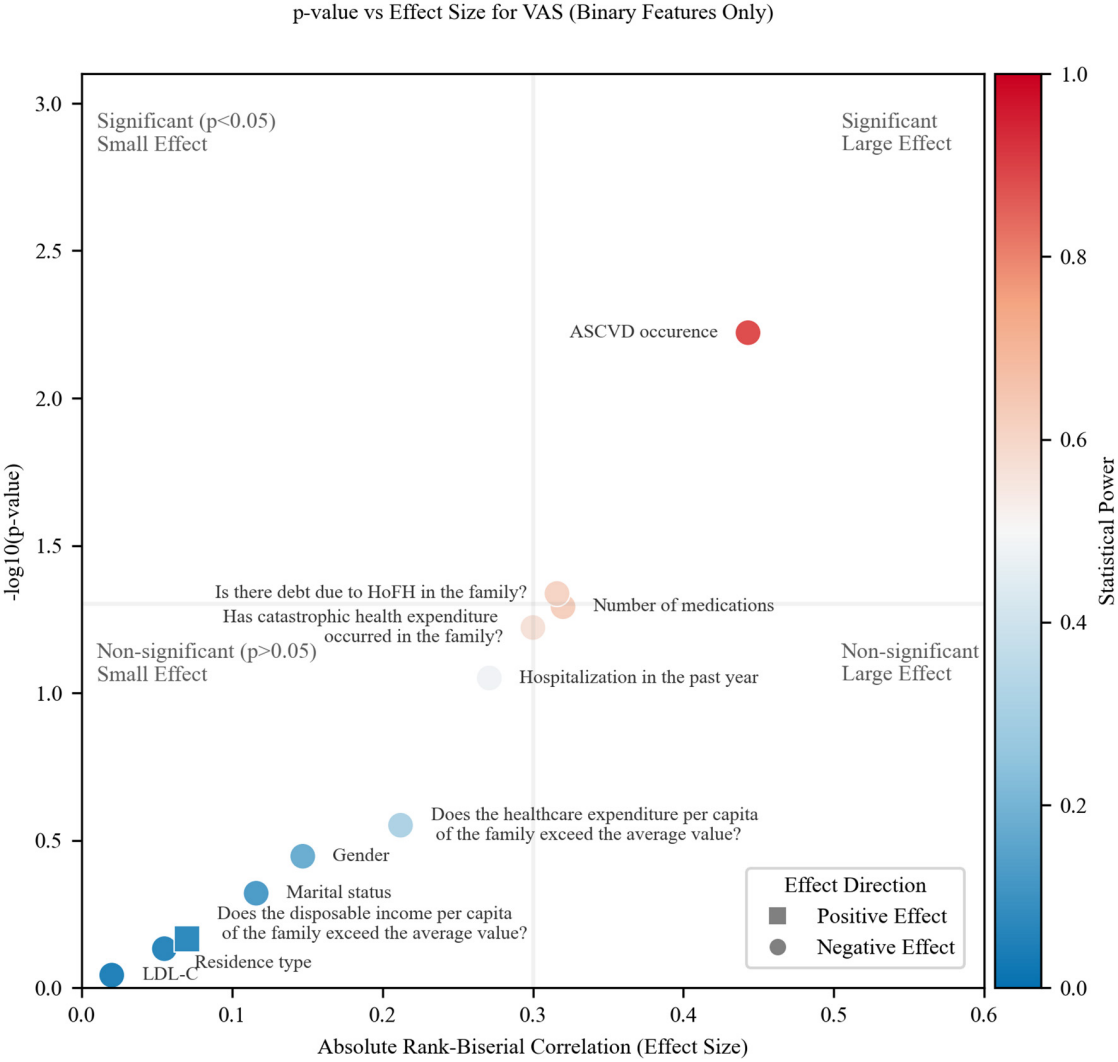
Effect size, significance and statistical power for binary features and VAS.

The VAS results (Figure 3) exhibit a similar quadrant distribution pattern to health utility, with two notable distinctions: most points cluster toward the bottom-left quadrant (non-significant, small effects), suggesting reduced statistical discriminability for VAS in this study; two factors—catastrophic health expenditure and number of medications, transition to the bottom-right quadrant, positioned near both threshold lines.

This systematic drift implies that VAS may be a less sensitive metric than health utility in the experimental context, as evidenced by: weaker overall associations (effect sizes) and fewer statistically significant findings despite comparable effect magnitudes for some features.

In contrast to the binary features, preliminary analysis of multiclass features revealed no significant effects (all ε^2^ < 0.06, *p* > 0.05, power < 0.1) for health utility or VAS outcomes; further details are omitted for brevity.

## 4 Discussion

This study analyzed the quality of life of HoFH patients through a screening program conducted at Beijing Anzhen Hospital. The quality of life for HoFH patients, particularly severe HoFH patients, is suboptimal. The factors negatively impact the quality of life of HoFH patients includes the occurrence of ASCVD, hospitalization in the past year, and taking multiple medication, weather healthcare expenditure per capita exceeding the average, catastrophic health expenditure, and debt due to HoFH. Among the considered factors, only the occurrence of ASCVD and debt due to HoFH can pass Benjamini-Hochberg procedure (α = 0.05), thus there is a solid association between health utility and the two factors above. These two factors demonstrated both statistical significance (all adjusted *p* < 0.05) and substantial relevance, as evidenced by large effect sizes (absolute rank-biserial correlation≈0.5) and adequate statistical power (>80%).

The mean utility of Chinese HoFH patients (0.849) was higher than the HoFH patients in the Netherlands (0.783) (28). Possible explanations include a higher mean age and more severe symptoms among the Dutch sample. Moreover, our study revealed higher utilities than those of other Chinese rare disease patients [0.659 (29) and 0.53(30)]. The latter sample included patients with advanced-stage rare diseases, whereas ours did not. Previous publications indicate that utility scores among different rare disease patients vary, such as those of cystic fibrosis patients in Europe, which range from 0.640 to 0.870 (31); familial hypercholesterolemia patients in the Netherlands (0.869) (32); and multiple system atrophy patients in China (0.558) (33). The mean utility for Chinese HoFH patients was found to be significantly lower than that of the general population [0.951 (34), 0.985 (35)] and lower than that of patients with hypertension (0.88) and stroke (0.90) (36). Furthermore, severe HoFH patients (those who experienced ASCVD or had LDL-C values exceeding 10 mmol/L) had a mean utility of only 0.819, which was lower than that of patients with common diseases, such as coronary heart disease (0.87) and diabetes (0.84) (36).

Our research has revealed that HoFH has a significant negative impact on employment, as less than half of these patients were employed full-time and a substantial proportion of patients (22.64%) were unemployed. These findings are consistent with previous studies (19, 37). The diminished employment rate among patients diagnosed with HoFH can be attributed to extended periods of treatment and heightened severity of the disease. Interestingly, we found that employed HoFH patients had higher mean and median utility scores than unemployed patients, which is in line with a previous study in China (38). This can be attributed to the fact that employment provides patients with a sense of social engagement, interaction with others, and a subjective sense of physical health. Additionally, higher quality of life may also lead to a higher employment rate for patients (38). Therefore, policymakers should take into consideration the importance of early diagnosis and treatment for HoFH patients, as well as creating suitable job opportunities to improve their employment prospects.

A study in Turkey found that patients with HoFH scored significantly worse than the general population in almost all domains of quality of life. Our study partly echoes these findings. We found that the percentage of patients with reported health problems in the dimensions of mobility, self-care, usual activities, pain/discomfort, and anxiety/depression was higher than that of the general population in China. However, pain/discomfort was the most commonly reported health problem in both HoFH patients and the general population (34, 35). Notably, our study documented the presence of anxiety or depression among HoFH patients; however, the small sample size limited further analysis. The available research on the association of HoFH with incident anxiety or depression is scarce, a point corroborated by a systematic review on HoFH and health-related quality of life (39). Therefore, expanding the sample size is a necessary next step to definitively examine this link.

To enhance the ability to diagnose and treat rare diseases in China, a joint effort from the National Health Commission and relevant departments resulted in the publication of two batches of the Rare Disease Catalog, encompassing a total of 207 diseases (40). This catalog explicitly defines the scope of rare diseases in China. In 2019, the “Rare Disease Diagnosis and Treatment Guidelines (2019 Edition)” (41) were published based on the “First Batch of Rare Disease Catalogs,” and a national rare disease diagnosis and treatment collaboration network was established. In October 2019, Peking Union Medical College Hospital developed the “China Rare Disease Diagnosis and Treatment Service Information System” (42), requiring 324 collaborating hospitals to provide real-time information on patients from the Rare Disease Catalog. By 2024, the number of hospitals in the national rare disease diagnosis and treatment collaboration network has expanded to 419, covering all provinces in China, significantly improving the management and treatment of rare diseases. These policy implementations have increased the standardization of rare disease diagnosis and treatment in China, but the system is still in its early stages. Research has shown that rare diseases in China have a long time from onset to diagnosis and high rates of misdiagnosis. A cross-sectional survey conducted in 2020 found that, on average, it took 4.81 years for 2,040 rare disease patients to receive a diagnosis, with over two-thirds experiencing misdiagnosis (43). This study found that the misdiagnosis rate among HoFH patients reached 83.02%, with 52.83% of HoFH patients being misdiagnosed for over 5 years. There are still issues of insufficient awareness and inadequate diagnosis and treatment in the HoFH (44). The delayed diagnosis and treatment of patients can accelerate disease progression, reduce quality of life, and impose a significant disease burden on both patients and their families during the period of misdiagnosis.

In recent years, the PCSK9 inhibitor evolocumab, the small interfering RNA cholesterol-lowering drug inclisiran, and two innovative oral fixed-dose combinations—ezetimibe/atorvastatin (II) and rosuvastatin/ezetimibe (I)—have been approved for the treatment of patients with HoFH. With the exception of inclisiran, the other three drugs have been included in the National Reimbursement Drug List, providing multiple treatment options for HoFH patients (45). Although we observed that statins remain the primary treatment for HoFH patients, the implementation of China's national volume-based procurement policy has significantly reduced the prices of statins. Additionally, the three new drugs have been included in the National Reimbursement Drug List through price negotiation, which will effectively improve the accessibility and affordability of medications for HoFH patients. Research indicates that the earlier the initiation of LA, the better the prognosis for patients. However, LA is expensive, which is why the rate of blood lipid purification therapy among Chinese HoFH patients is very low. Only 2 out of 53 HoFH patients have used LA, accounting for 3.77%. Consequently, debt due to HoFH emerges as one of the most significant determinants. Therefore, it is emphatically suggested that policymakers should intensify the advancement, promotion, accessibility, and availability of interventions related to HoFH. Additionally, they should also consider provision of financial assistance for patients suffering from HoFH.

Given that HoFH impacts a minuscule fraction of the population, the understanding of these patients' quality of life remains significantly limited among healthcare practitioners and the broader public. To our knowledge, this study is the first to comprehensively investigate the quality of life and its determinants in HoFH patients in China. Another contribution lies in the orchestration of robust statistic methods, such as multiple test correction, effect size and post-hoc power analysis, which are employed to minimize type I error and to counteract the limitations posed by small sample sizes typical in rare disease study. Moreover, we believe that our research will lead to a better understanding of how best to support patients with HoFH. However, there are a few limitations that need to be taken into account in this study. First, the participants were HoFH patients who visited the outpatient clinic of Beijing Anzhen Hospital or attended free clinics across the country, which may cause potential geographical bias. The health utility scores for HoFH patients seem somewhat better compared to QoL findings in three studies which have assessed QoL in HoFH (19, 28, 37), since patients had to travel to these locations, those with limited mobility and self-care may not be included in the study, which could have led to higher utility values for HoFH patients than the actual patients' group. Second, out of the 53 HoFH patients, 25 patients (47.17%) selected the highest possible utility value, indicating a serious ceiling effect when measuring utility values for HoFH patients using the EQ-5D. Third, this project was designed as an ongoing investigation, but was interrupted by China's epidemic prevention and control policy from 2020 to 2022. Therefore, survey data from 2017 to 2019 were used for our data analysis, where it could be argued that the data may be outdated. Nevertheless, we believe that quality of life resembles a stable indicator of clinical effectiveness that does not change significantly over time. This suggests that our study remains valuable and informative despite the interruption. Additionally, due to the unavailability of an EQ-5D-Y value set for patients under the age of 12 in China when the questionnaire was designed, only patients above the age of 12 were included, resulting in a relatively small sample size for this research, despite the small population of HoFH patients. Finally, the EQ-5D is a widely used generic measure of health-related quality of life that has been validated in many different patient populations. However, its appropriateness for use in HoFH patients remains unclear.

## 5 Conclusions

This study investigated the quality of life of HoFH patients in China and explored the factors that influence the quality of life of HoFH patients. The quality of life of HoFH patients is lower than that of the general population. Factors which demonstrated negative impact (unadjusted *p* < 0.05) on the quality of life of HoFH patients include the occurrence of ASCVD, hospitalisation in the past year, taking multiple medications, higher than average healthcare expenditure per capita, catastrophic healthcare expenditure and debt due to HoFH. The result of Benjamini-Hochberg procedure reveals that the occurrence of ASCVD and debt due to HoFH have the most significant association and substantial relevance with health utility. Policy makers should consider the policy that improves early diagnosis, employment prospects, and the availability of HoFH-related interventions and provision of financial assistance for patients suffering from HoFH.

## Data Availability

The raw data supporting the conclusions of this article will be made available by the authors, without undue reservation.
