# Supplementary material for: Quality of life and its contributors among patients with homozygous familial hypercholesterolemia in China
Source: Front Public Health. 2025 Aug 27;13:1642895. doi: 10.3389/fpubh.2025.1642895 (PMC12421902; doi:10.3389/fpubh.2025.1642895)
Supplement: Supplementary file 1 [file Table_1.docx]

# **Supplementary Material**

**Table S1 Statistical test assumptions checking results for health utility measures**

| **Feature** | **Group** | **n** | **Shapiro-Wilk p** | **Levene p** | **Test** |
| --- | --- | --- | --- | --- | --- |
| Gender | Male | 26 | <0.001 | 0.331 | Mann-Whitney U |
|  | Female | 27 | 0.001 |  |  |
| Age | 12-19 | 12 | 0.003 | 0.324 | Kruskal-Wallis |
|  | 20-29 | 19 | <0.001 |  |  |
|  | ≥30 | 22 | 0.003 |  |  |
| Residence type | Rural | 30 | <0.001 | 0.849 | Mann-Whitney U |
|  | Urban | 23 | <0.001 |  |  |
| Marital status | Unmarried | 32 | <0.001 | 0.196 | Mann-Whitney U |
|  | Married | 21 | 0.002 |  |  |
| Employment status | Employed | 23 | <0.001 | 0.961 | Kruskal-Wallis |
|  | Unemployed | 12 | 0.124 |  |  |
|  | Student | 18 | 0.002 |  |  |
| Diagnosis time | 0-4 years | 24 | <0.001 | 0.968 | Kruskal-Wallis |
|  | 5-9 years | 13 | <0.001 |  |  |
|  | ≥10 years | 16 | 0.098 |  |  |
| Disease duration | 0-9 years | 16 | <0.001 | 0.343 | Kruskal-Wallis |
|  | 10-19 years | 21 | 0.002 |  |  |
|  | ≥20 years | 16 | 0.025 |  |  |
| ASCVD occurrence | No | 23 | <0.001 | 0.003 | Mann-Whitney U |
|  | Yes | 30 | 0.005 |  |  |
| LDC-C | ≤10 mmol/L | 23 | <0.001 | 0.839 | Mann-Whitney U |
|  | >10 mmol/L | 30 | <0.001 |  |  |
| Hospital in the past year | No | 27 | <0.001 | 0.016 | Mann-Whitney U |
|  | Yes | 26 | 0.003 |  |  |
| Number of medications | <5 | 33 | <0.001 | 0.083 | Mann-Whitney U |
|  | ≥5 | 20 | 0.022 |  |  |
| Does the disposable income per capita of the family exceed the average value? | No | 35 | <0.001 | 0.138 | Mann-Whitney U |
|  | Yes | 18 | 0.002 |  |  |
| Does the healthcare expenditure per capita of the family exceed the average value? | No | 11 | <0.001 | <0.001 | Mann-Whitney U |
|  | Yes | 42 | <0.001 |  |  |
| Has catastrophic health expenditure occurred in the family? | No | 29 | <0.001 | 0.059 | Mann-Whitney U |
|  | Yes | 24 | 0.016 |  |  |
| Is there debt due to HoFH in the family? | No | 27 | <0.001 | 0.022 | Mann-Whitney U |
|  | Yes | 26 | 0.035 |  |  |

**Table S2 Statistical test assumptions checking results for VAS measures**

| **Features** | **Groups** | **n** | **Shapiro-Wilk p** | **Levene p** | **Test** |
| --- | --- | --- | --- | --- | --- |
| Gender | Male | 26 | 0.044 | 0.236 | Mann-Whitney U |
|  | Female | 27 | 0.012 |  |  |
| Age | 12-19 | 12 | 0.048 | 0.619 | Kruskal-Wallis |
|  | 20-29 | 19 | 0.154 |  |  |
|  | ≥30 | 22 | 0.291 |  |  |
| Residence type | Rural | 30 | 0.010 | 0.927 | Mann-Whitney U |
|  | Urban | 23 | 0.051 |  |  |
| Marital status | Unmarried | 32 | 0.009 | 0.771 | Mann-Whitney U |
|  | Married | 21 | 0.111 |  |  |
| Employment status | Employed | 23 | 0.045 | 0.426 | Kruskal-Wallis |
|  | Unemployed | 12 | 0.014 |  |  |
|  | Student | 18 | 0.032 |  |  |
| Diagnosis time | 0-4 years | 24 | 0.065 | 0.302 | Kruskal-Wallis |
|  | 5-9 years | 13 | 0.174 |  |  |
|  | ≥10 years | 16 | 0.032 |  |  |
| Disease duration | 0-9 years | 16 | 0.031 | 0.883 | Kruskal-Wallis |
|  | 10-19 years | 21 | 0.384 |  |  |
|  | ≥20 years | 16 | 0.118 |  |  |
| ASCVD occurrence | No | 23 | 0.037 | 0.230 | Mann-Whitney U |
|  | Yes | 30 | 0.143 |  |  |
| LDC-C | ≤10 mmol/L | 23 | 0.015 | 0.783 | Mann-Whitney U |
|  | >10 mmol/L | 30 | 0.088 |  |  |
| Hospital in the past year | No | 27 | 0.031 | 0.442 | Mann-Whitney U |
|  | Yes | 26 | 0.229 |  |  |
| Number of medications | <5 | 33 | 0.019 | 0.308 | Mann-Whitney U |
|  | ≥5 | 20 | 0.153 |  |  |
| Does the disposable income per capita of the family exceed the average value? | No | 35 | 0.019 | 0.409 | Mann-Whitney U |
|  | Yes | 18 | 0.327 |  |  |
| Does the healthcare expenditure per capita of the family exceed the average value? | No | 11 | 0.119 | 0.771 | Mann-Whitney U |
|  | Yes | 42 | 0.046 |  |  |
| Has catastrophic health expenditure occurred in the family? | No | 29 | 0.028 | 0.223 | Mann-Whitney U |
|  | Yes | 24 | 0.243 |  |  |
| Is there debt due to HoFH in the family? | No | 27 | 0.010 | 0.134 | Mann-Whitney U |
|  | Yes | 26 | 0.345 |  |  |

**Table S3 Non-parametric statistical results for health utility**

| **Features** | **Categories** | **p-value** | **Effect Size** | **Effect Size Type** | **Current Power** | **Required N** |
| --- | --- | --- | --- | --- | --- | --- |
| Gender | 2 | 0.228 | -0.184 | r_rb_ | 0.255 | 118 |
| Age | 3 | 0.412 | 0.000 | ε² | NA | inf |
| Residence type | 2 | 1.000 | -0.001 | r_rb_ | 0.050 | 1868423 |
| Marital status | 2 | 0.336 | -0.150 | r_rb_ | 0.186 | 175 |
| Employment status | 3 | 0.351 | 0.002 | ε² | 0.050 | 2732008 |
| Diagnosis time | 3 | 0.290 | 0.009 | ε² | 0.050 | 107805 |
| Disease duration | 3 | 0.175 | 0.030 | ε² | 0.051 | 10867 |
| ASCVD occurrence | 2 | 0.001 | -0.499 | r_rb_ | 0.941 | 17 |
| LDC-C | 2 | 0.367 | -0.139 | r_rb_ | 0.166 | 204 |
| Hospital in the past year | 2 | 0.037 | -0.318 | r_rb_ | 0.613 | 40 |
| Number of medications | 2 | 0.029 | -0.342 | r_rb_ | 0.678 | 35 |
| Does the disposable income per capita of the family exceed the average value? | 2 | 0.371 | 0.144 | r_rb_ | 0.175 | 190 |
| Does the healthcare expenditure per capita of the family exceed the average value? | 2 | 0.010 | -0.485 | r_rb_ | 0.929 | 18 |
| Has catastrophic health expenditure occurred in the family? | 2 | 0.016 | -0.368 | r_rb_ | 0.739 | 30 |
| Is there debt due to HoFH in the family? | 2 | 0.001 | -0.493 | r_rb_ | 0.936 | 18 |

Abbreviations: r_rb_= rank biserial correlation; ε² = epsilon-squared

**Table S4 Non-parametric statistical results for VAS**

| **Feature** | **Categories** | **p-value** | **Effect Size** | **Effect Size Type** | **Current Power** | **Required N** |
| --- | --- | --- | --- | --- | --- | --- |
| Gender | 2 | 0.358 | -0.147 | r_rb_ | 0.180 | 184 |
| Age | 3 | 0.652 | 0.000 | ε² | NA | inf |
| Residence type | 2 | 0.737 | -0.055 | r_rb_ | 0.068 | 1295 |
| Marital status | 2 | 0.478 | -0.116 | r_rb_ | 0.130 | 293 |
| Employment status | 3 | 0.677 | 0.000 | ε² | NA | inf |
| Diagnosis time | 3 | 0.310 | 0.007 | ε² | 0.050 | 204394 |
| Disease duration | 3 | 0.116 | 0.046 | ε² | 0.052 | 4497 |
| ASCVD occurrence | 2 | 0.006 | -0.443 | r_rb_ | 0.880 | 21 |
| LDC-C | 2 | 0.906 | -0.020 | r_rb_ | 0.052 | 9534 |
| Hospital in the past year | 2 | 0.089 | -0.271 | r_rb_ | 0.482 | 55 |
| Number of medications | 2 | 0.051 | -0.320 | r_rb_ | 0.618 | 40 |
| Does the disposable income per capita of the family exceed the average value? | 2 | 0.683 | 0.070 | r_rb_ | 0.078 | 806 |
| Does the healthcare expenditure per capita of the family exceed the average value? | 2 | 0.281 | -0.212 | r_rb_ | 0.323 | 89 |
| Has catastrophic health expenditure occurred in the family? | 2 | 0.060 | -0.300 | r_rb_ | 0.565 | 45 |
| Is there debt due to HoFH in the family? | 2 | 0.046 | -0.316 | r_rb_ | 0.609 | 41 |

†NA = Not applicable when effect size equals zero (ε² = 0), “inf” indicates theoretically infinite sample size required

‡ Abbreviations: r_rb_= rank biserial correlation; ε² = epsilon-squared

**Tabel S5 Geographic distribution of HoFH patients across provinces in China**

| **Province** | **Numbers** |
| --- | --- |
| Anhui | 9 |
| Hebei | 5 |
| Jiangsu | 5 |
| Shanxi | 5 |
| Zhejiang | 5 |
| Fujian | 3 |
| Henan | 3 |
| Sichuan | 3 |
| Gansu | 2 |
| Hubei | 2 |
| Jiangxi | 2 |
| Shandong | 2 |
| Guizhou | 1 |
| Heilongjiang | 1 |
| Jilin | 1 |
| Liaoning | 1 |
| Ningxia | 1 |
| Shaanxi | 1 |
| Chongqing | 1 |
| Total | 53 |
